# Characterisation of microRNA expression in post-natal mouse mammary gland development

**DOI:** 10.1186/1471-2164-10-548

**Published:** 2009-11-20

**Authors:** Stefanie Avril-Sassen, Leonard D Goldstein, John Stingl, Cherie Blenkiron, John Le Quesne, Inmaculada Spiteri, Konstantina Karagavriilidou, Christine J Watson, Simon Tavaré, Eric A Miska, Carlos Caldas

**Affiliations:** 1 Breast Cancer Functional Genomics Laboratory, Cancer Research UK Cambridge Research Institute and Department of Oncology, University of Cambridge, Li Ka Shing Centre, Cambridge, UK; 2 Computational Biology Group, Cancer Research UK Cambridge Research Institute and Department of Oncology, University of Cambridge, Li Ka Shing Centre, Cambridge, UK; 3 Mammary Stem Cell Biology Group, Cancer Research UK Cambridge Research Institute, Li Ka Shing Centre, Cambridge, UK; 4 Wellcome Trust/Cancer Research UK Gurdon Institute and Department of Biochemistry, University of Cambridge, Cambridge, UK; 5 Computational Biology Group, Department of Applied Mathematics and Theoretical Physics, University of Cambridge, Cambridge, UK; 6 Department of Pathology, University of Cambridge, Cambridge, UK; 7 Current address: Department of Pathology, Technische Universität München, Munich, Germany

## Abstract

**Background:**

The differential expression pattern of microRNAs (miRNAs) during mammary gland development might provide insights into their role in regulating the homeostasis of the mammary epithelium. Our aim was to analyse these regulatory functions by deriving a comprehensive tissue-specific combined miRNA and mRNA expression profile of post-natal mouse mammary gland development.

We measured the expression of 318 individual murine miRNAs by bead-based flow-cytometric profiling of whole mouse mammary glands throughout a 16-point developmental time course, including juvenile, puberty, mature virgin, gestation, lactation, and involution stages. In parallel whole-genome mRNA expression data were obtained.

**Results:**

One third (n = 102) of all murine miRNAs analysed were detected during mammary gland development. MicroRNAs were represented in seven temporally co-expressed clusters, which were enriched for both miRNAs belonging to the same family and breast cancer-associated miRNAs. Global miRNA and mRNA expression was significantly reduced during lactation and the early stages of involution after weaning. For most detected miRNA families we did not observe systematic changes in the expression of predicted targets. For miRNA families whose targets did show changes, we observed inverse patterns of miRNA and target expression. The data sets are made publicly available and the combined expression profiles represent an important community resource for mammary gland biology research.

**Conclusion:**

MicroRNAs were expressed in likely co-regulated clusters during mammary gland development. Breast cancer-associated miRNAs were significantly enriched in these clusters. The mechanism and functional consequences of this miRNA co-regulation provide new avenues for research into mammary gland biology and generate candidates for functional validation.

## Background

MicroRNAs (miRNAs) are a class of small non-coding RNA, approximately 23 nucleotides in length, which regulate gene expression post-transcriptionally and play a key role in development and specific biological processes such as cell proliferation, differentiation, and apoptosis [1-3]. MicroRNAs regulate their target genes through direct degradation of the messenger RNA (mRNA) and/or translational inhibition [4-7]. While subsets of miRNAs are specifically expressed during early mammalian development [8], others are essential for morphogenesis of particular organs, such as brain [9] or heart [10], or hematopoietic differentiation [11,12]. In specific mammalian cell types or organs single tissue-specific miRNAs have been identified [13,14]. There is little information available regarding miRNA expression in the mammary gland. In human breast tissue, 23 miRNAs (out of 161 studied) were identified by microarray analysis [15] and 9 miRNAs (out of 22) were detected in mouse mammary gland by northern blot [16].

**Figure 1:**
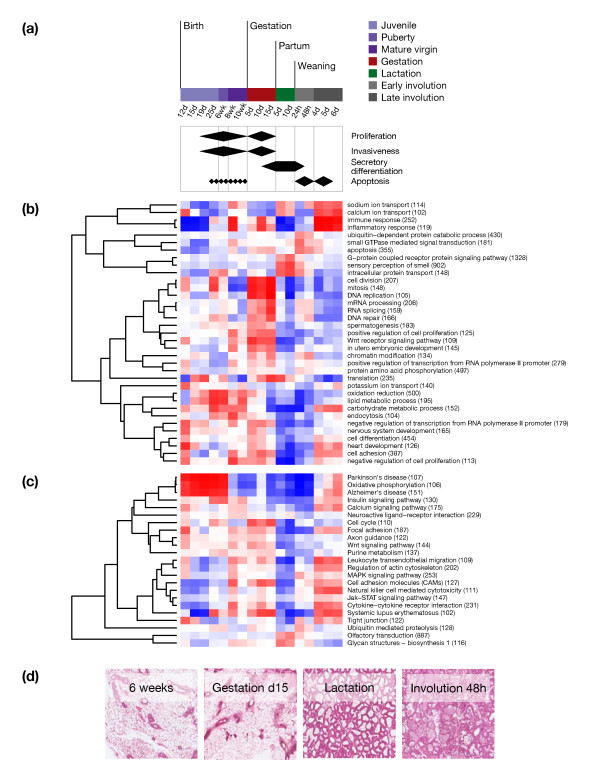
**Normal development of the mouse mammary gland**. (a) Schematic of distinct stages of development and characteristic biological processes. Shown are time points used for mRNA expression profiling (time point 12 hours involution was used for miRNA expression profiling only, time points 8 weeks and 10 days lactation were used for mRNA expression profiling only). (b-c) Mean relative log_2 _mRNA expression for non-redundant Gene Ontology biological processes (b) and KEGG pathways (c) (shown are gene sets with at least 100 genes represented on the array, numbers of genes are indicated in brackets). Red and blue indicate high and low relative log_2 _expression, respectively. (d) Representative H&E stained histological sections of mouse mammary glands obtained at distinct time points during development and used for expression analysis.

Mammary gland development in both humans and mice is predominantly a post-natal process (Figure 1a). At birth, rudimentary mammary ducts are present, derived from the ectodermal mammary placode. Ovarian and pituitary hormones induce ductal outgrowth and branching during puberty, at about three to six weeks of age in mice, followed by alveolar differentiation during gestation and full functional differentiation upon parturition and lactation. After weaning, the entire alveolar compartment is remodelled to resemble a virgin-like state. Each gestation initiates a new round of lobulo-alveolar differentiation [17,18]. These morphologic changes are illustrated in Figure 1d. Our aim was to study the pattern and temporal changes of miRNA expression during mouse mammary gland development. In parallel, gene expression data were obtained to provide another view of the underlying biological processes at the mRNA level.

Altered miRNA expression has been implicated in various human diseases including cancer [19]. In human breast cancer, complex de-regulated miRNA expression patterns have been described [20-23]. The expression of 29 miRNAs was significantly different between human normal breast tissue and breast cancer [21], and a panel of 38 miRNAs was found to be differentially expressed between molecular subtypes of breast cancer, namely Luminal A, Luminal B, Her-2 positive, Basal-like, and Normal-like breast cancers [20]. This suggests that miRNAs might play a role in regulating the homeostasis of the mammary epithelial hierarchy and their disruption could therefore contribute to tumourigenesis.

The mammary gland is unique in its capability to undergo cycles of cell proliferation, differentiation, and apoptosis during adult life. More importantly, the mammary epithelium invades the stroma during mammary gland development in a process similar to that of cancer. Therefore we investigated the miRNA expression pattern during mammary gland development to provide the basis for comparisons with breast cancer profiles and for selection of representative miRNA candidates for future functional characterization in breast development and breast cancer.

## Results

### mRNA expression during mammary gland development

Our use of whole glands (after carefully removing the mammary lymph node) for RNA isolation could raise questions as to whether the expression changes reflect epithelial-driven biological processes. To gain an overview of changes in mRNA expression between different developmental stages we assessed the activity of biological processes and pathways as determined by the mean relative expression of genes belonging to the same Gene Ontology term or KEGG pathway (Figure 1b, c). In accordance with previous reports, we found that most developmental stages were dominated by a few biological processes and pathways which are known to be involved in regulating mammary epithelial biology. For example during puberty and gestation, proliferation and associated processes of cell division and mitosis were highly represented, calcium/sodium ion transport, translation and intracellular protein transport were prominent during lactation, apoptosis dominated during early involution and inflammatory/immune response was dominant during late involution (Figure 1b). We further assessed the proportion of luminal, basal, and stromal cells at eight developmental time points up to mid-gestation by fluorescence-activated cell sorting (FACS) of dissociated total mouse mammary glands. The proportion of stromal cells, which comprised up to 90% of the whole gland in juvenile stages, gradually declined until puberty, staying relatively constant thereafter comprising 65%-75% of the mammary gland. The proportion of luminal cells showed an increase before the onset of puberty with a peak at mid-puberty, followed by a slow decrease during gestation. Basal cells showed a slow and steady increase after the onset of puberty (Additional file 1). Expression levels of luminal (Krt18) and basal (Krt14) cytokeratins reflected the changes in the epithelial compartment (Additional file 2). Although stromal cells contribute significant numbers of cells to the mammary gland, the mRNA expression data show that the observed differential gene expression mostly reflected mammary epithelium-driven processes. This also suggests the observed miRNA differential expression is mostly associated with mammary epithelium-driven events.

### MicroRNA expression profiles distinguish the stages of mammary gland development

**Figure 2:**
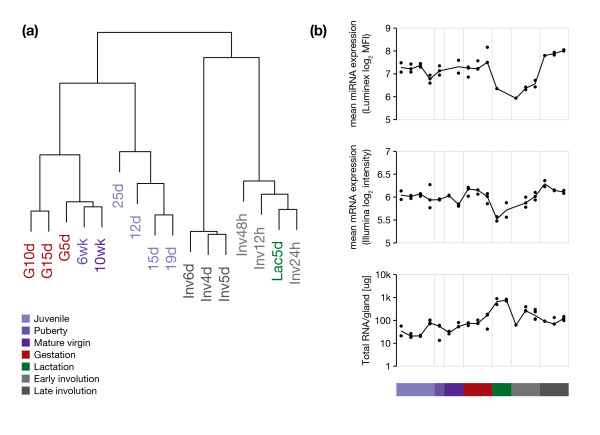
**Global changes in miRNA and mRNA expression**. (a) Hierarchical clustering of time points based on the relative log_2 _expression of 102 detected miRNAs. (b) miRNAs and mRNAs exhibit reduced overall expression during lactation and early involution time points. Black dots correspond to independent biological replicates. Median values for each time point are connected by a solid black line. (Top) miRNA expression. Normalised log_2 _median fluorescence intensity (MFI) values (see Methods) were averaged for each sample by taking the arithmetic mean. (Middle) mRNA expression. The background corrected Illumina probe level data were transformed by taking logs (base 2) but no between sample normalisation was performed. Non-normalised log_2 _intensities were averaged for each sample by taking the arithmetic mean. (Bottom) Mass of total RNA extracted from whole mammary glands, and equally sized partial glands for lactation and involution time points.

Out of 318 unique murine miRNAs analysed, 102 miRNAs were detected above background in one or more developmental time points. The majority of miRNAs were expressed intermittently throughout development and were not present in one or more developmental stages. We first asked whether the global miRNA expression profiles of time points would allow us to separate developmental stages. Unsupervised hierarchical clustering of time points based on relative miRNA expression resulted in a clear separation of developmental stages, with close clustering of time points with chronological or functional dependence (Figure 2a). The early juvenile time points (12, 15, 19, 25 days) clustered together, as did the consecutive stages puberty (6 weeks), mature virgin (10 weeks), and early gestation (gestational day 5). Lactation and the early stages of involution (between 12 and 48 hours after weaning) clustered together and were clearly separated from the cluster of late involution stages, between 4 and 6 days after weaning. Profiles based on the expression of 102 miRNAs thus distinguished distinct stages of mammary gland development in a similar manner to mRNA expression signatures (Additional file 3).

### Global decrease in miRNA and mRNA expression during lactation and early involution

During lactation and the early stages of involution (12, 24 and 48 hours after weaning) we observed an approximately two-fold reduction in mean miRNA expression (*P *< 0.01, two-sided Wilcoxon rank sum test; Figure 2b). In contrast, overall miRNA expression was increased approximately two-fold during the late stages of involution (4, 5 and 6 days after weaning) as compared to earlier developmental time points (*P *< 0.01, two-sided Wilcoxon rank sum test). No obvious differences in global miRNA expression were detected between the remaining time points, including juvenile, virgin, and gestation stages (Figure 2b). A similar decrease was observed for the number of miRNAs present at a given time point. During lactation and early involution, an average of 47 individual miRNAs were detected at each time point (range 38 to 56), compared to an average of 70 miRNAs present at other time points during development (range 55 to 87). Changes in miRNA expression during lactation and early involution were paralleled by a decrease in mean mRNA expression as assessed by non-normalised microarray expression data (*P *< 0.05, two-sided Wilcoxon rank sum test). In contrast to the decrease in miRNA and mRNA expression during lactation and early involution, we observed a dramatic increase in the total RNA content per mammary gland for these time points (Figure 2b).

### MicroRNAs are expressed in seven distinct temporal clusters during development

**Figure 3:**
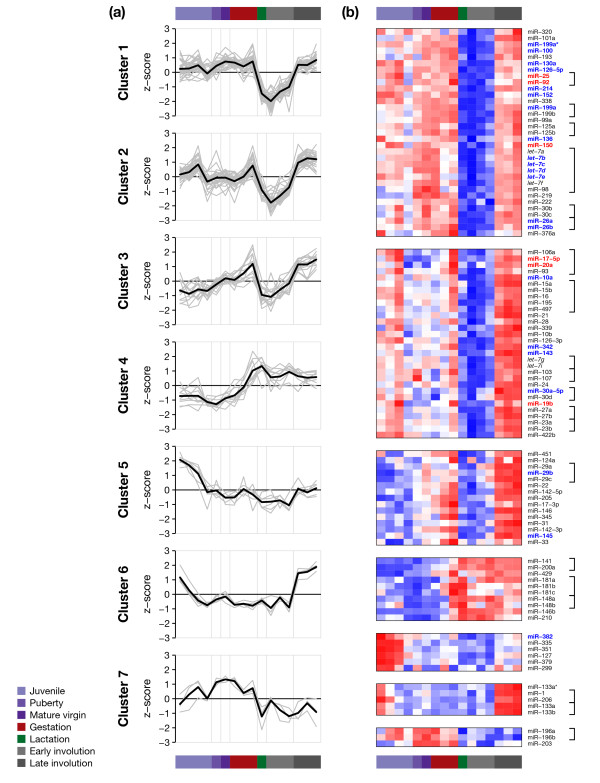
**miRNA expression during mammopoiesis is highly correlated**. Seven clusters obtained by model-based clustering are displayed in separate panels. (a) Grey lines represent the standardized log_2 _expression profiles of individual miRNAs. Black lines correspond to mean cluster profiles. (b) Heatmaps of individual clustered miRNAs. Red and blue indicate high and low standardized log_2 _expression, respectively. Within each cluster panel, miRNAs are ordered according to miRNA families with identical seed sequence (position 2-8) (indicated by brackets). miRNAs associated with basal or luminal breast cancer are highlighted in red and blue, respectively. Individual panels correspond to the seven clusters in (a).

We next aimed to identify groups of miRNAs with similar relative expression patterns over the course of mammary gland development. Model-based clustering [24] identified seven clusters with distinct temporal expression profiles (Figure 3), and most individual miRNA profiles were assigned to one of the seven clusters with high certainty (84 with certainty > 0.99, minimum certainty 0.76).

The three largest clusters (clusters 1-3) showed a decrease in miRNA expression during lactation and early involution, but differed in their typical expression at earlier time points. While many miRNAs in cluster 1, including six members of the *let-7 *family, showed high expression during puberty (six weeks), maturity (ten weeks) and early gestation (five days), members of cluster 2 frequently peaked in their expression before puberty (19 days) and during late gestation (15 days). Cluster 3 miRNAs generally showed a monotone increase in expression over time, reaching maximum expression in late gestation (15 days). In stark contrast to the typical pattern of reduced miRNA expression during lactation and early involution, cluster 4 showed highest expression during these stages of development. This cluster included the seed-identical miRNAs miR-141 and miR-200a, and miR-429, which is situated in the same genomic cluster as miR-200a. It further included miR-146b, miR-210 and multiple members of the miR-148 and miR-181 families. Clusters 5 and 6 exhibited very distinct profiles, with the former being highly expressed exclusively in early development. Cluster 6, containing the miR-1/206 and miR-133 family, showed a distinct peak of expression during late involution, between 4 and 6 days after weaning, as well as a second smaller peak during early development from 12 to 15 days of age. The five miRNAs temporally co-expressed in this expression cluster are also clustered in the genome (at two distinct loci on chromosomes 1 and 18). The profile of cluster 7, including miR-196a/b and miR-203, showed an inverse pattern to cluster 4, with high expression in early development and gestation followed by low expression in lactation and involution stages.

miRNAs in the same cluster often shared identical seed sequences (indicated by brackets in the right-hand margin of Figure 3b) and thus likely share common mRNA targets. An analysis of biological processes and pathways enriched or depleted among predicted cluster targets is given in Additional file 4. We also observed genomic clustering of many co-expressed miRNAs (Additional file 5), suggesting polycistronic transcription.

### Breast cancer-associated miRNAs during mammary gland development

We next assessed whether orthologs of the miRNAs that had previously been associated with various clinicopathologic and molecular parameters of human breast cancer were represented within the seven temporal clusters of miRNAs during mammary gland development. The selection of breast cancer-associated miRNAs was based on data that we previously reported [20] (see Methods).

Interestingly, miRNAs with increased expression in luminal as compared to basal breast cancers (see Methods) were often co-expressed during development, and strikingly enriched in cluster 1 (*P *< 0.001, two-sided Fisher's exact test), with 14 out of 21 miRNAs identified as highly expressed in luminal breast cancers representing more than 40% of cluster 1 miRNAs. In contrast, miRNAs overexpressed in the basal breast cancer subtype (four members of the miR-17~92 family, miR-25 and miR-150) were distributed equally among clusters 1 and 2 (Figure 3b).

### MicroRNA expression in distinct cell types assessed by in-situ hybridisation

**Figure 4:**
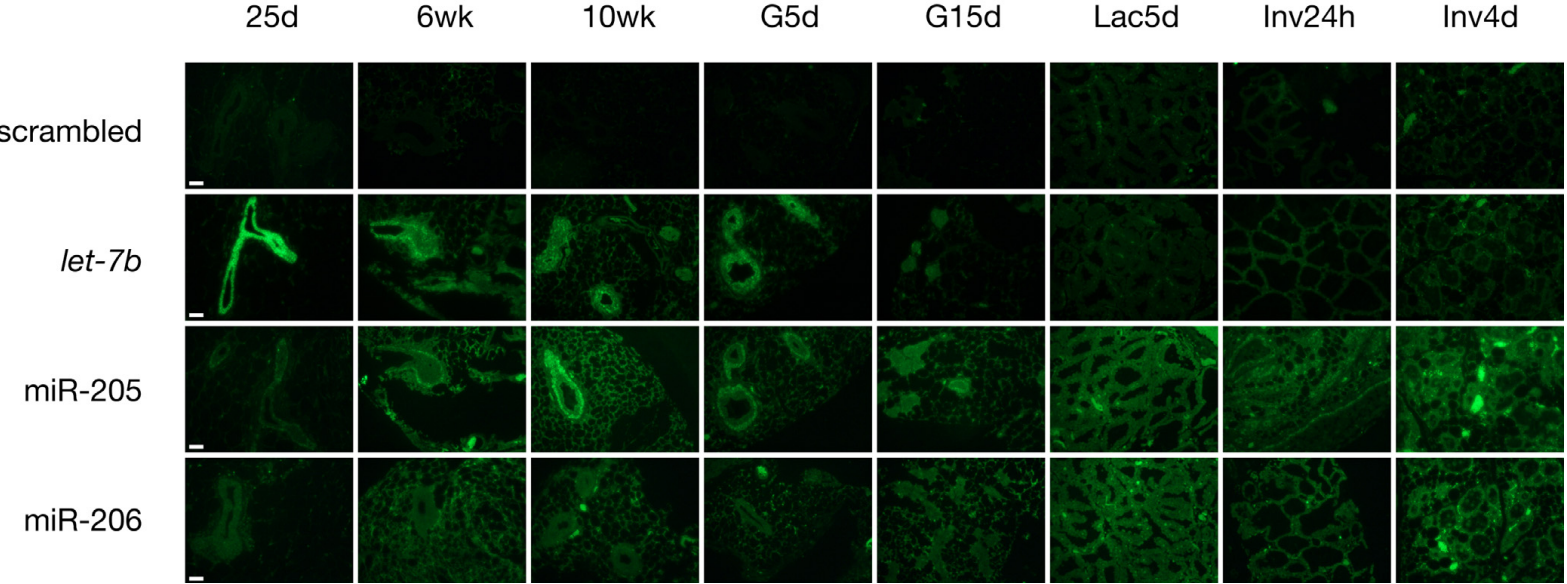
**In-situ hybridisation for miRNAs let-7b (cluster 1), miR-205 (cluster 3), and miR-206 (cluster 6) shows higher expression in the epithelial compared to the stromal cell compartment**. let-7b and miR-205 show specificity for the luminal and basal epithelial cell layers, respectively. White scale bars in images for time point 25d indicate 100 microns. Higher magnification images are provided in Additional file 6.

To further assess cellular origin of miRNA expression, we analysed representative miRNAs from three out of the seven clusters by in-situ hybridisation (Figure 4 and Additional file 6). There was generally much higher expression of all miRNAs analysed in the epithelial cells compared to the stroma. The background signal from stromal tissue showed high variability between different probes, and a random pattern of staining. Although this suggests non-specific binding in the stroma, weak miRNA expression cannot be excluded. The strongest background staining of stromal tissue was found in the developmental stages of lactation and involution.

Overall, there was good agreement between expression patterns found by in-situ hybridisation and by microarray. Two miRNAs, namely let-7b and miR-205, showed specificity for the luminal and basal epithelial cell layers, respectively, during juvenile, puberty, and mature virgin stages. For let-7b (cluster 1), luminal expression was lost during pregnancy, lactation and early involution, and expression recurred in both luminal and basal epithelium in late involution. miR-205 (cluster 3) was strongly expressed in the basal epithelium until the mature virgin stage, showing increased expression in both luminal and basal epithelium during pregnancy and in late involution. miR-206 (cluster 6) showed increasing expression during late involution, in accordance with the microarray data. Weak expression compared to background was present in juvenile and gestational time points, which was more pronounced in the microarray data. Detailed in-situ images for all developmental stages are shown in Figure 4.

### Altered expression of miRNA target genes during mammary gland development

**Figure 5:**
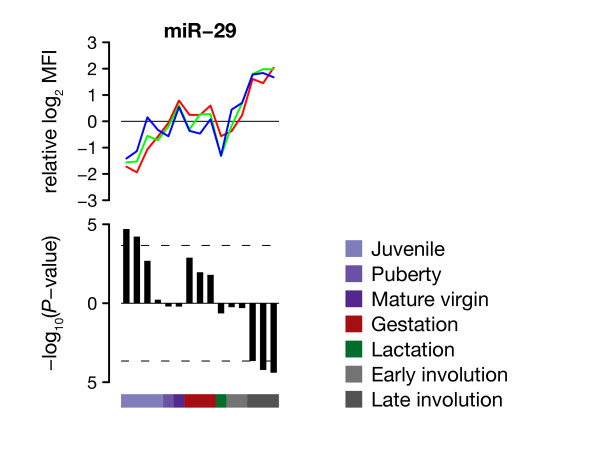
**Predicted targets for the miR-29 family show systematic changes in their relative expression**. (Top) Relative log_2 _expression profiles of individual miR-29 family members are shown in distinct colours. (Bottom) Negative Log_10 _transformed two-sided *P*-values are shown for each time point. The positive and negative y-axes indicate increased and reduced mean relative expression levels compared to control genes, respectively. Dashed lines indicate the threshold for Benjamini-Hochberg corrected *P*-values smaller than 0.05.

To investigate systematic and specific changes in the expression of miRNA targets, the 102 detected miRNAs were grouped in families with unique seed sequence (nucleotides 2-8) and 3' UTRs of genes with available expression data were scanned for seed matches to identify putative targets [25-29]. For each of these miRNA families and each developmental time point, we tested whether the mean relative expression of putative targets was higher or lower than that of cohorts of control genes with similar 3' UTR features (see Methods). For most miRNA families we did not detect any significant changes (Additional file 7). Figure 5 shows results for the miR-29 family, which showed greatest evidence for changes in the expression of predicted targets (*P *< 0.05 after Benjamini-Hochberg correction). Low relative expression of miR-29 during early development and high expression during late involution were paralleled by increased and reduced relative expression of seed match-harbouring transcripts, respectively. Among putative miR-29 targets genes related to focal adhesion were significantly overrepresented (Additional file 4).

## Discussion

Our data show that miRNAs are highly co-regulated during mammary gland development. We found seven temporal clusters with complex expression patterns which did not coincide with single developmental stages. Breast cancer-associated miRNAs were found predominantly in two of these clusters.

MicroRNAs previously identified to be highly expressed in the luminal as compared to the basal molecular subtype of breast cancer [20] were enriched in cluster 1. These miRNAs showed high levels during puberty and gestation where proliferation and invasion are the predominating biological processes. We hypothesize that these luminal breast cancer miRNAs may be involved in the control of proliferation and invasion during normal development and become deregulated in breast cancer. MicroRNAs associated with the basal breast cancer subtype, including four members of the miR-17~92 family, miR-25 and miR-150, showed a distinctly different pattern, being equally distributed among clusters 1 and 2.

Of particular interest was cluster 4, which in contrast to the global decrease in miRNA expression during lactation and involution showed a specific increase in expression during these stages, paralleled by a decrease in the expression of predicted targets for one of the cluster members, miR-429 (Additional file 7). The miRNAs included in this cluster have previously been associated with various biological functions and pathways. The cluster includes miR-200 family members miR-200a, miR-141 and miR-429. There is increasing evidence that this miRNA family plays a crucial role in the regulation of epithelial to mesenchymal transition (EMT). All five members of the miR-200 family were markedly down-regulated in cells that had undergone EMT in response to transforming growth factor (TGF)-beta [30]. Enforced expression of the miR-200 family was sufficient to prevent TGF-beta induced EMT, while inhibition of the miR-200 family was sufficient to induce EMT in cells [30]. During mammary gland development, a process similar to EMT is known to occur between lactation and involution. The loss of inter-epithelial cell-cell-contacts, including loss of the cell-adhesion molecule E-cadherin, takes place during the early reversible phase of involution [31]. It is therefore tempting to speculate that the miR-200 family may act to prevent premature loss of E-cadherin and induction of EMT during lactation.

Another miRNA highly expressed during lactation and early involution, miR-146b, has been associated with the regulation of innate immune response and inflammation. miR-146a expression has been reported to be substantially increased in murine T helper (Th) 1 cells compared to Th2 cells and naïve T cells [32]. The Th1 and Th2 cytokine milieu has recently been implicated in mouse mammary gland development. Mammary epithelial cells were demonstrated to undergo a switch from Th1 to Th2 cytokine production upon the induction of luminal differentiation [33]. It is an intriguing possibility that miR-146 might be involved in abrogating the Th2 bias in cytokine expression that continues during gestation and lactation, to facilitate a switch back to a Th1 environment upon involution.

Of note, we did not detect obvious changes in the miRNA expression pattern between the stages of lactation and early involution in our data, although there are known morphological and transcriptional changes [34-37]. This contrasts with what we observed at the level of mRNA expression, where we could see clear differences between the two stages (Figure 1b, c). Subtle differences in miRNA expression between lactation and early involution may have potentially been masked by the global decrease in miRNA expression we observed during these stages.

We identified a significant global down-regulation of miRNAs and mRNAs during the stages of lactation and early involution. The total RNA content per mammary gland was increased during these stages. Since our analysis of miRNA expression was performed in equal amounts of total RNA, this finding suggests that miRNAs and mRNAs represented a smaller fraction of total RNA during lactation and early involution. We hypothesize that the up-regulation of the transcriptional and translational machinery during milk production (with corresponding increases in tRNAs/rRNAs) may lead to a relative rather than absolute depletion of miRNAs during these developmental stages.

During mouse mammary gland development we observed that many *let-7 *family members showed a peak in expression during puberty, the mature virgin stage, and early gestation, followed by a marked decrease and low levels during lactation and involution. This observation is consistent with recent reports that let-7 expression is depleted in mouse mammary epithelial progenitors [38] and in breast tumour-initiating cells [39], and that enforced let-7 expression could inhibit the self-renewal capacities of cells [38,39]. The developmental stages of the juvenile gland, puberty, and early gestation, where we detected increasing levels of let-7, are characterised by a marked expansion of the luminal and alveolar cell compartment which presumably contains a high frequency of relatively differentiated cells. The subsequent decrease in let-7 expression during lactation may allow for a relative expansion of the progenitor compartment which is necessary for the reconstitution of the alveolar compartment during the next pregnancy cycle. On the other hand, miR-22 and miR-205, which were reported to be highly expressed in mammary progenitor cells [38], seemed to be enriched during gestation and again during late involution. These data suggest that miRNAs may contribute to the balance between progenitor cells and their differentiated progeny during mammary gland development.

When interpreting changes in miRNA expression during mammary gland development, changes in the cellular composition of the mammary gland have to be taken into consideration. We observed that the proportion of stromal cells gradually declined until puberty. Epithelial and in particular luminal cells showed a peak at puberty followed by a decrease, which was reflected in gene expression patterns, such as those of luminal and basal cytokeratins. Nevertheless, the variety of biological processes and pathways and their observed temporal expression pattern (Figure 1b, c) cannot be solely explained by changes in the cellular composition. Although stromal cells contribute significant numbers of cells to the mammary gland, our mRNA expression data show that the observed differential gene expression mostly reflected mammary epithelium-driven processes, and therefore we infer that changes in miRNA expression are also predominantly epithelial-driven. We were not able to demonstrate by microarray analysis a clear correlation between the expression of a particular miRNA and a predominating cell type. It is an inherent limitation of this study that the impact of cell type heterogeneity on relative expression patterns obtained from whole mammary glands cannot be conclusively assessed. Future analysis of isolated cell populations may help to address this question, and newer technologies for miRNA profiling which have become available in the meantime allow the analysis of smaller numbers of cells. Nevertheless, our analysis of three candidate miRNAs by in-situ hybridisation gives some indication of cell type specific expression in mammary epithelial cells. MicroRNA expression was generally much higher in epithelial cells compared to the stroma, suggesting that the expression patterns found by microarray mostly reflect temporal changes in the epithelial cell compartment. Within the epithelial compartment, cell type-specific expression was demonstrated for let-7 and miR-205, which were predominantly expressed in luminal and basal cells, respectively, confirming previous reports of their expression in human luminal and basal breast epithelium [40].

It was shown previously that miRNAs and their targets often exhibit mutually exclusive expression both in the spatial and temporal domain [41], and that conserved targets and miRNAs are co-expressed but with reduced relative target expression in the presence of the miRNA [7]. We failed to detect widespread systematic changes in the relative expression of putative targets during mammary gland development. However, we did observe trends for individual miRNA families (Additional file 7), most notably the miR-29 family, for which miRNAs and targets showed anti-correlated expression (Figure 5). These observations are consistent with miRNAs affecting mRNA expression levels [7,42]. The down-regulation of miR-29 targets, enriched for focal adhesion genes, would fit with the extensive remodelling and changes during late involution. Future analysis of isolated cell populations will further contribute to investigations of the relationship between miRNAs and their targets.

## Conclusion

We expect that this first comprehensive data set of miRNA and mRNA gene expression during mammary gland development will provide an important resource for the future functional characterisation of individual miRNAs and their targets in mammary epithelial cells. Indeed with our current understanding of miRNA target recognition (see [43] for a recent review) combined data sets such as the one presented here represent important resources that will have increasing value as data integration methods improve. It is likely that with further functional experimentation new miRNA target-recognition rules will be uncovered and these data will also be an important functional genomics resource for their biological validation.

## Methods

### Mammary gland samples

#### Tissue collection

Six- to ten-week old female C57Bl/6 mice were obtained from Charles River Laboratories (Margate, UK) and either culled for virgin mammary glands or mated at 10 weeks of age. For involution time points females were force weaned at 10 days of lactation. Pups were culled at the specified age for juvenile time points. Tissue was always collected at the same time of day to account for circadian effects. Mammary glands of the following six key developmental stages, including 18 time points, were harvested: juvenile (12, 15, 19, and 25 days of age), puberty (6 weeks of age), mature virgin (8, 10 weeks of age), gestation (gestational day 5, 10, and 15), lactation (day 5, 10 post partum), early involution (12 h, 24 h, 48 h after weaning), and late involution (4 d, 5 d, and 6 d after weaning). A total of 2 to 3 animals, designated biological replicates, were used per time point. In each case one complete inguinal gland was removed following excision of the intra-mammary lymph node and frozen in liquid nitrogen within 30 seconds after tissue dissection. The contralateral abdominal glands of each animal were immediately fixed in 4% formaldehyde overnight and embedded in paraffin according to standard procedures for morphological analysis. Frozen samples were stored at -80°C and paraffin embedded samples were stored at room temperature, for 1 to 10 months until further analysis. Whole mounts of mammary glands were prepared for time points until late gestation using Carnoy's fixative and Carmine Alum stain, to allow for a three-dimensional visualization of mammary ducts and lobules. All experimental research on animals was carried out according to UK Home Office regulations.

#### RNA extraction

Frozen tissue samples were homogenized in Trizol reagent (Invitrogen, Carlsbad, CA, USA) with a polytron tissue homogenizer, using a whole mammary gland per sample for juvenile, virgin, and gestational time points, and equally sized partial glands for lactation and involution time points. After pre-spinning homogenized samples at 10,000 rpm at 4°C for 10 minutes to remove fat and cell debris, total RNA was extracted from the supernatant according to the manufacturer's protocol (Invitrogen, Carlsbad, CA, USA), modified by washing the final RNA pellet in 80% ethanol. RNA quantity and integrity were confirmed with a NanoDrop spectrophotometer (NanoDrop Technologies, Wilmington, DE, USA) and Agilent 2100 bioanalyser (Agilent Technologies, Santa Clara, CA). Only samples with an RNA integrity number ≥ 7 were used for further miRNA expression analysis, and only samples with an RNA integrity number ≥ 8 were used for gene (mRNA) expression analysis.

#### Analysis of cellular composition of mammary glands

Flow cytometry was used to analyse the cellular composition of mammary glands at all eight time points up to mid-gestation [44]. Mammary glands were digested for 8 h at 37°C in EpiCult-B with 5% fetal bovine serum (FBS), 300 U/ml collagenase and 100 U/ml hyaluronidase. After vortexing and lysis of the red blood cells in NH_4_Cl, a single cell suspension was obtained by sequential dissociation of the fragments by gentle pipetting for 1-2 min in 0.25% trypsin, and then 2 min in 5 mg/ml dispase II plus 0.1 mg/ml DNase I (DNase; Sigma Aldrich, St. Louis, MO, USA) followed by filtration through a 40-μm mesh (BD, Franklin Lakes, NJ, USA). All reagents were from StemCell Technologies Inc (Vancouver, BC, Canada) unless otherwise specified.

The cells were then incubated with a monoclonal antibody specific for CD49f (clone GoH3 from Biolegend, San Diego, CA, USA) that was conjugated to Alexa Fluor-488. The cells were also incubated with a monoclonal antibody specific for epithelial cell adhesion molecule (EpCAM; clone G8.8 from BD) that was previously conjugated to Alexa Fluor-647 (Invitrogen). Biotin-conjugated antibodies specific for CD45, Ter119 and CD31 (clones 30-F11, Ter119 and 390, respectively; all from eBioscience, San Diego, CA, USA) were used to identify and exclude contaminating hematopoietic and endothelial cells. Streptavidin-conjugated phycoerythrin Texas-Red (Pharmingen) was used to detect biotin conjugated antibodies. Propidium iodide (Sigma) was used at 1 μg/ml for live/dead cell discrimination. The proportion of luminal, basal, and stromal cells was determined by analysing the cells on a CyAn™ ADP (Beckman Coulter, Fullerton, CA, USA). Stromal, luminal and basal subpopulations were identified by their CD24^-^, CD24^high ^CD49f^low ^and CD24^low ^CD49f^high ^phenotypes, respectively [44]

#### MicroRNA in-situ hybridisation

Paraffin embedded mammary gland tissue blocks were assembled into a tissue microarray including 2-4 cores of 2 mm diameter per sample. Sections of 4 micrometer thickness were deparaffinized in xylene and rehydrated in an ethanol dilution series, followed by proteinase K digestion (30 μg/ml for 15 min at 37°C). After refixation in 4% paraformaldehyde, slides were prehybridised in hybridisation solution (50% formamide, 0.1% Tween, 50 μg/ml heparin, 5× SSC, 500 μg/ml yeast tRNA, pH 6) at 50-70°C for 30 min. miRCURY™ LNA-modified DNA oligonucleotides complementary to the miRNAs of interest were purchased pre-labelled with digoxigenin at the 5' and 3' ends (Exiqon Inc., Vedbaek, Denmark). Slides were hybridised with 100 μl of hybridisation solution containing 5 pmol of DIG-labelled LNA probe overnight at 50-70°C. After stringency washes (two rinses in 5 × SSC, followed by 3 washes of 20 min in 50% formamide/2 × SSC at the hybridisation temperature, with agitation), sections were rinsed 5 times in PBS/0.1%Tween-20 (PBST), and blocked for 1 hour in blocking solution (2% sheep serum, 2 mg/ml BSA in PBST). LNA probes were detected using Roche horseradish peroxidase-conjugated anti-digoxigenin Fab fragments (Roche Diagnostics, Mannheim, Germany). An amplified fluorescent signal was then obtained using the Perkin-Elmer TSA Plus tyramide signal amplification kit (Perkin Elmer, MA, USA). For each LNA probe, all images were obtained from one array slide using the same settings on a Nikon confocal microscope, and signals were visually quantified.

### MicroRNA expression analysis

MicroRNA isolation and labelling was performed as described previously [20]. In brief, for each sample small RNAs (18-26 nucleotides) were recovered from 5 μg total RNA through denaturing polyacrylamide gel purification, after spiking in three synthetic pre-labelling control RNAs to account for target preparation efficiency. Small RNAs were adaptor-ligated sequentially on the 3'-end and 5'-end using T4 RNA ligase (Fermentas, Burlington, ONT, Canada). After reverse-transcription using adaptor-specific primers, products were PCR-amplified using a biotinylated 5'-primer.

The expression of 318 unique murine miRNAs was determined using a bead-based flow-cytometric microarray platform as previously described [20,45]. The platform was adapted to include 334 probes for 318 unique murine miRNAs based on the miRNA repository miRBase 9.1 [46] as well as probes for six artificial control RNAs. In brief, oligonucleotide capture probes complementary to the miRNAs of interest were conjugated to colour coded polystyrene beads (xMAP beads, Luminex Corporation, Austin, TX, USA) allowing the simultaneous detection of 90 different target miRNAs. To obtain expression profiles for 318 miRNAs, four distinct sets of bead-coupled oligonucleotide probes were created including replicate probes, and each sample was hybridised to all four bead sets. Samples were hybridised to probes in solution overnight at 50°C, including technical replicate samples as well as two water-only blanks and three bead blanks containing water instead of the labelled sample for use as a background control. After washing off unbound sample, the sample-bead solution hybrids were incubated with streptavidin-phycoerythrin at 50°C for 10 min to detect sample-bound biotin moieties. Median fluorescence intensity values were measured on a Luminex 100IS machine using StarStation software (ACS, Sheffield, UK).

MicroRNA expression data were submitted to the NCBI Gene Expression Omnibus http://www.ncbi.nlm.nih.gov/geo and are available under accession number GSE15057.

### Gene expression analysis

Whole genome mRNA expression analysis was performed using Sentrix Mouse-6 Expression BeadChips V1 (Illumina Inc., San Diego, CA, USA). For each sample, 200 ng of total RNA were labelled using the Illumina TotalPrep RNA Amplification Kit according to the manufacturer's instructions (Applied Biosystems, Foster City, CA, USA). Labelled samples were hybridised to BeadChips overnight (18 hours) at 55°C and stringency washes were performed according to the manufacturer's protocol. Data were acquired using Illumina BeadArray Reader and BeadScan software (Illumina Inc., San Diego, CA, USA).

Gene expression data were submitted to the NCBI Gene Expression Omnibus http://www.ncbi.nlm.nih.gov/geo and are available under accession number GSE15057.

### Quantitative real-time PCR

Expression of 6 selected miRNAs, including let-7b, let-7g, miR-1, miR-146, miR-16, and miR-17-5p, was validated using TaqMan MicroRNA Assays according to the manufacturer's instructions (Applied Biosystems, Foster City, CA, USA) and normalising data to U6 small nuclear RNA (RNU6; Applied Biosystems) expression. Real-time PCR reactions and data acquisition were performed on the ABI Prism 7900HT sequence detection system (Applied Biosystems). Details are summarized in Additional file 8.

Expression values of 6 selected genes (mRNA), including Foxa1, Gata3, Krt14, Krt18, Stat3, and Stat5a, were validated by quantitative reverse transcription real-time polymerase chain reaction (qRT-PCR) using custom designed exon-spanning primers and SYBR Green reporter, normalising data to the expression values of beta-actin (Actb) and cyclophilin A (Ppia) as housekeeping genes (Additional data file 2).

### Computational data analysis

#### Preprocessing of miRNA expression data

Median fluorescence intensities smaller than 1 were set equal to 1 and all values were transformed by taking logs (base 2). For each sample, two controls spiked into total extracted RNA were required to exceed a mean intensity of 12 on the log_2 _scale. 40 out of 57 samples (70%) passed this quality control. To filter out probes for absent miRNAs, each probe was required to exceed 6 on the log_2 _scale in all replicate samples for one or more time points. The criterion was satisfied by 118 probes, corresponding to 102 unique miRNAs. Technical sample effects were estimated by mean relative levels of the two spike-in controls (one measurement per bead set and control, eight measurements in total) and each sample was normalised by subtracting the estimated technical effect. Four probes replicated within and between bead sets and three pairs of technical replicate samples were summarized by their mean expression profiles. Biological replicate samples were summarized by their median expression profiles.

#### Preprocessing of mRNA expression data

Illumina Mouse v1 gene expression data were processed and summarized in the Illumina BeadStudio software. Analysis of the background corrected probe level data was performed using the *beadarray *Bioconductor package [47]. Data were transformed by taking logs (base 2) and quantile normalised [48]. Four pairs of technical replicate samples were summarized by their mean expression profiles. Biological replicate samples were summarized by their median expression profiles.

#### Hierarchical clustering

Hierarchical clustering of median summarized time points was performed with Pearson correlation and average linkage. All probes were centred to have mean zero before clustering.

#### Clustering of miRNA profiles

Clusters of miRNAs were identified using the MCLUST package [24]. miRNA profiles over 16 median summarized time points were centred and scaled to have mean zero and standard deviation one. A multivariate normal mixture model was fitted to the data, imposing spherical clusters with identical variance, and allowing for up to 20 mixture components. A model with seven mixture components achieved maximal Bayesian Information Criterion.

#### Breast cancer-associated miRNAs

Human miRNAs associated with basal and luminal breast cancers were those differentially expressed between 16 basal-like and 15 luminal A samples in [20] (Benjamini-Hochberg adjusted *P *< 0.05, two-sided Wilcoxon rank sum test). Mouse orthologs were identified based on sequence identity of the mature miRNA sequence.

#### Target prediction

Mouse 3' UTR sequences were obtained from the TargetScan website (Release 5.1) [49]. The 102 detected miRNAs were grouped in 65 families with identical seed sequence (nucleotides 2-8). Since the annotation of many miRNAs was revised after the beginning of the study (miRBase Release 9.1), we only considered 58 miRNA families whose seed sequence was represented among mouse miRNAs in miRBase Release 13.0. Targets for individual miRNA families were defined as genes with at least one perfect 3' UTR seed match. Targets for a given miRNA cluster were defined as genes with at least one perfect 3' UTR match to the seed sequence of any of the miRNAs included in that cluster. For each 3' UTR we calculated the probability of one or more random matches to the seed sequence of an individual miRNA family and to those represented in a given cluster using a compound Poisson approximation for a second-order Markov model [50].

#### Target expression

Preprocessed intensities for Illumina probes with unambiguous RefSeq annotation for genes with available 3' UTR information were extracted from the mRNA expression data. Multiple probes assigned to the same RefSeq ID were summarized by their mean expression profile. Biological replicate samples for the same time point were summarized by their median expression profile, and the summarized expression profile for each RefSeq gene was centred to have median zero.

For a given time point and miRNA family, the mean relative expression of predicted targets was compared to the mean relative expression of an identical number of control genes. 100,000 control cohorts were obtained by drawing an identical number of genes from the pool of all genes with available 3' UTR information and expression data, with the probability of an individual gene being selected proportional to the probability of it containing at least one seed match (see previous section). We obtained empirical *P*-values for reduced expression as the number of control cohorts with mean expression smaller than or equal to the one observed. Subsequently *P*-values were converted to two-sided *P*-values. Figure 5 and Additional file 7 show negative log transformed (base 10) two-sided *P*-values for those miRNA families with greatest evidence for change in the expression of their targets (based on the minimum observed *P*-value). The positive and negative y-axes correspond to increased and reduced expression, respectively. Dashed lines indicate the threshold for Benjamini-Hochberg corrected *P*-values smaller than 0.05 (correcting for 58 × 17 = 986 multiple tests).

All analyses were performed using custom Perl scripts and the statistical programming environment R [51] using customised functions and functions available from Bioconductor [52,53].

## Authors' contributions

SAS participated in conception and design of the study, carried out the provision of study materials and data acquisition, participated in data analysis and interpretation, and drafted the manuscript. LDG performed the data analysis and participated in data interpretation and manuscript writing. JS participated in the provision of study materials, data acquisition, data interpretation and manuscript writing. CB participated in the miRNA profiling by bead-based arrays. JLQ carried out the in-situ hybridisations. IS participated in the mRNA Illumina arrays. KK participated in the mRNA qPCR. CJW participated in the provision of study materials and data interpretation. ST participated in the data analysis, data interpretation and manuscript writing. EAM participated in the study conception and design, data interpretation, and manuscript writing. CC conceived of the study, participated in its design, data interpretation, and manuscript writing. All authors read and approved the final manuscript.

## Supplementary Material

Additional file 1 **Cellular composition of mouse mammary glands**. Changes in the cellular composition of the mammary gland are shown for eight developmental time points up to mid-gestation. The proportion of luminal, basal, and stromal cells was measured by fluorescence-activated cell sorting (FACS) of dissociated total mouse mammary glands. Click here for file

Additional file 2 **Validation of gene (mRNA) expression by qRT-PCR**. a. Validation of selected mRNA genes by qRT-PCR using custom designed primers and SYBR Green reporter (left panel), normalising data to the expression values of housekeeping genes beta-actin (Actb) and cyclophilin A (Ppia) (bottom rows). Expression data obtained by Illumina profiling (middle panel) showed good correlation with qRT-PCR data (right panel). b. Detailed description of SYBR Green qPCR according to MIQE guidelines. Click here for file

Additional file 3 **Hierarchical clustering of time points based on mRNA expression data**. Prior to clustering log_2 _intensities were mean centred across time points and multiple probes assigned to the same Entrez ID were summarized by their mean expression profile. Click here for file

Additional file 4 **Enrichment and depletion of GO biological processes (a, b) and KEGG pathways (c, d) among predicted miRNA targets**. Target genes were defined as genes with at least one 3' UTR match to the seed sequence of one or more miRNAs in a given cluster (a, c) or to individual seed-identical miRNA families (b, d). Log_2 _fold-enrichments are shown as heatmaps with red and green corresponding to enrichment and depletion, respectively. Significance was assessed by a two-sided Fisher's Exact test. Shown are gene sets with Benjamini-Hochberg corrected *P *< 0.01 (out of all statistically significant GO terms for a given cluster or miRNA family, only the most specific GO terms were included). Click here for file

Additional file 5 **miRNA expression and genomic location**. Heatmap as in main text Figure 3b with miRNAs in individual clusters reordered according to their genomic loci. Brackets in the right-hand margin indicate genomic clusters. A mature miRNA was assigned to a genomic cluster if its locus was situated within 50 kb of another cluster member (genomic clusters in distinct regions of the genome that shared a common mature miRNA were merged). Click here for file

Additional file 6 **Higher-magnification images of miRNA in-situ hybridisation**. Higher-magnification images of in-situ hybridisation for miRNAs let-7b, miR-205, and miR-206, showing higher expression in the epithelial compared to the stromal cell compartment, and specificity for the luminal and basal epithelial cell layers for let-7b and miR-205, respectively. White scale bars in images for time point 25d indicate 100 microns. Click here for file

Additional file 7 **Changes in the expression of predicted miRNA targets**. Negative Log_10 _transformed two-sided *P*-values (see Methods) are shown as heatmaps with red and green corresponding to evidence for increased and reduced relative expression levels, respectively. Target genes were defined as genes with at least one 3' UTR match to the seed sequence of one or more miRNAs in a given cluster (a) or to individual seed-identical miRNA families (b). miRNA families were ordered by the evidence for systematic changes in the expression of their targets (based on the minimum observed *P*-value). Asterisks indicate Benjamini-Hochberg corrected *P *< 0.05. (c) Greatest evidence for systematic changes in target expression was observed for miRNA families miR-29, miR-200bc/429 and *let-7*/miR-98/1961. Dashed lines indicate the threshold for Benjamini-Hochberg corrected *P*-values smaller than 0.05. Click here for file

Additional file 8 **Validation of miRNA expression by qRT-PCR**. a. Validation of selected miRNA genes by qRT-PCR using TaqMan miRNA Assays (left panel). qRT-PCR measurements were normalised to U6 small nuclear RNA (U6sRNA; bottom row). Expression data obtained by Luminex bead-based profiling (middle panel) showed good correlation with qRT-PCR data (right panel). b. Detailed description of TaqMan qPCR according to MIQE guidelines. Click here for file
