# Supplementary figures and images for: Characterisation of microRNA expression in post-natal mouse mammary gland development
Source: BMC Genomics. 2009 Nov 20;10:548. doi: 10.1186/1471-2164-10-548 (PMC2784809; doi:10.1186/1471-2164-10-548)

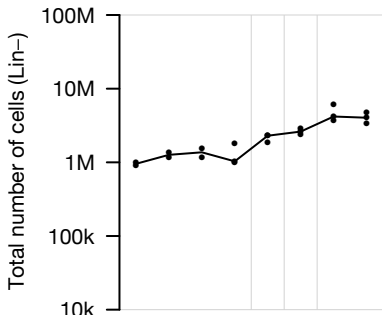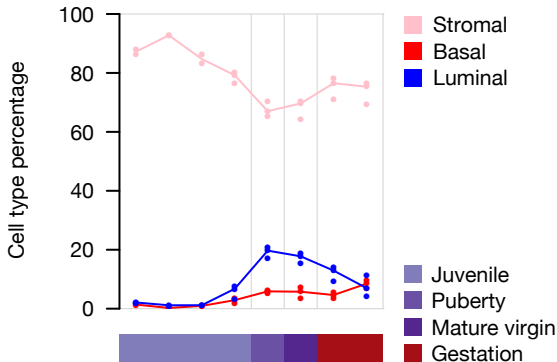

Supplement: Additional file 1 — Cellular composition of mouse mammary glands. Changes in the cellular composition of the mammary gland are shown for eight developmental time points up to mid-gestation. The proportion of luminal, basal, and stromal cells was measured by fluorescence-activated cell sorting (FACS) of dissociated total mouse mammary glands. [file 1471-2164-10-548-S1.PDF]

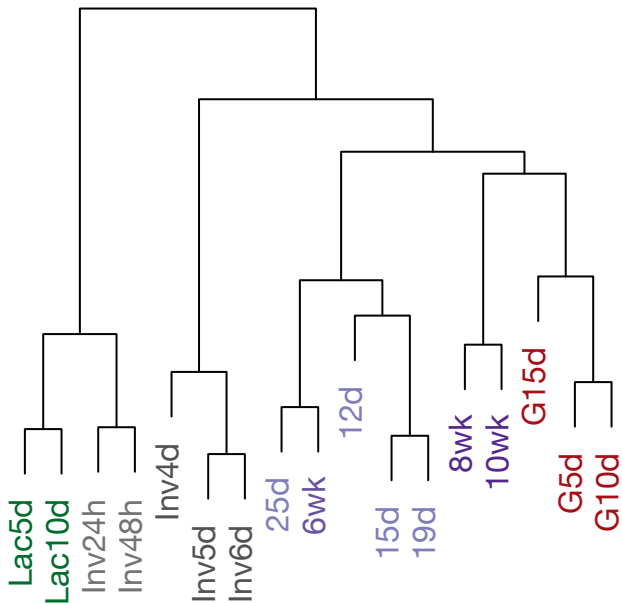

- Juvenile
- Puberty
- Mature virgin
- Gestation
- Lactation
- Early involution
- Late involution

Supplement: Additional file 3 — Hierarchical clustering of time points based on mRNA expression data. Prior to clustering log2 intensities were mean centred across time points and multiple probes assigned to the same Entrez ID were summarized by their mean expression profile. [file 1471-2164-10-548-S3.PDF]

(a)

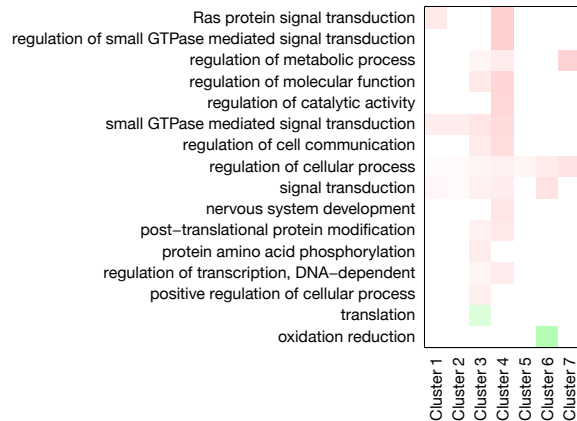

(b)

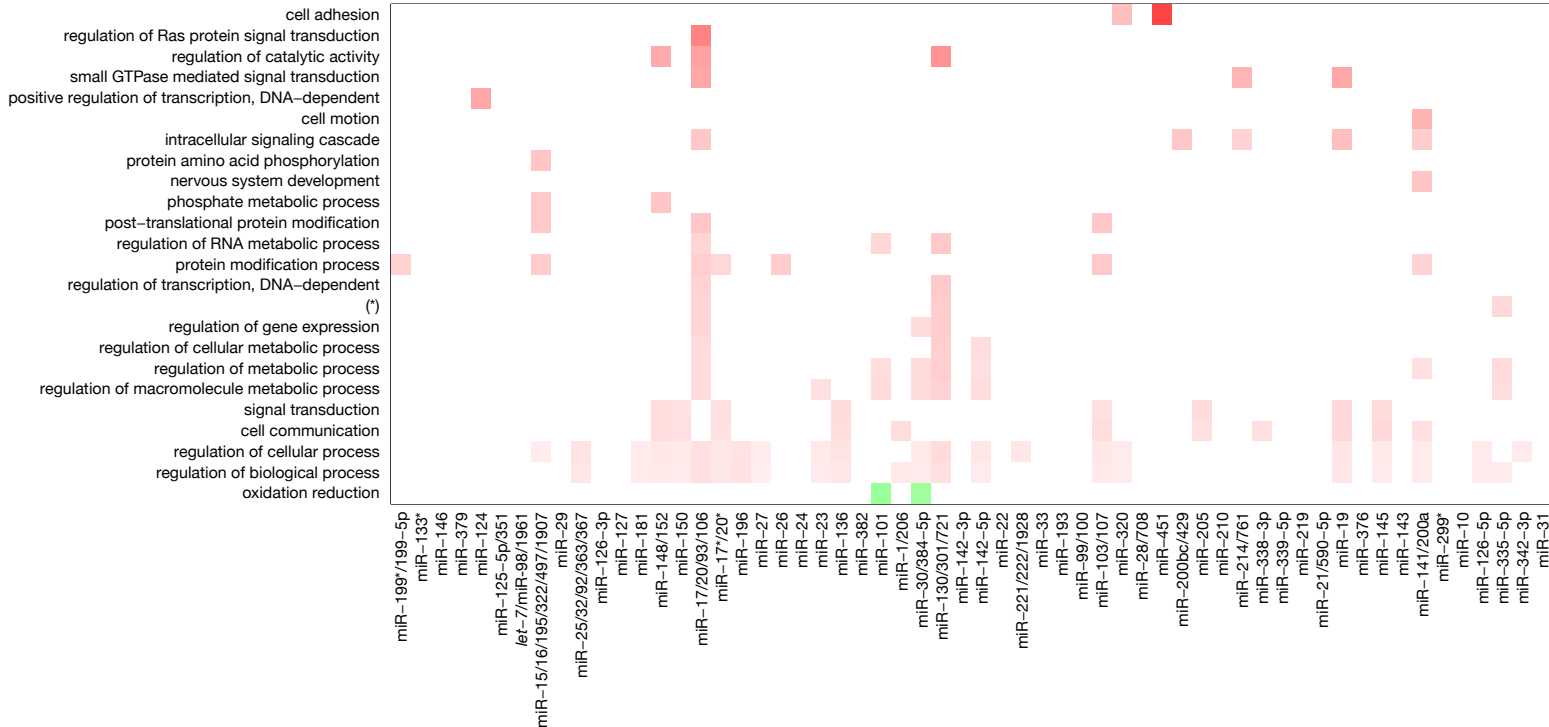

(c)

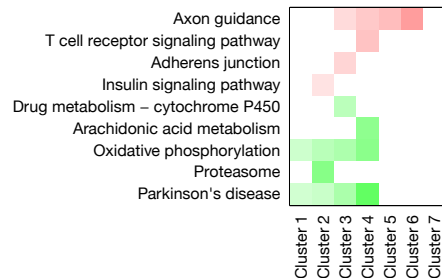

(d)

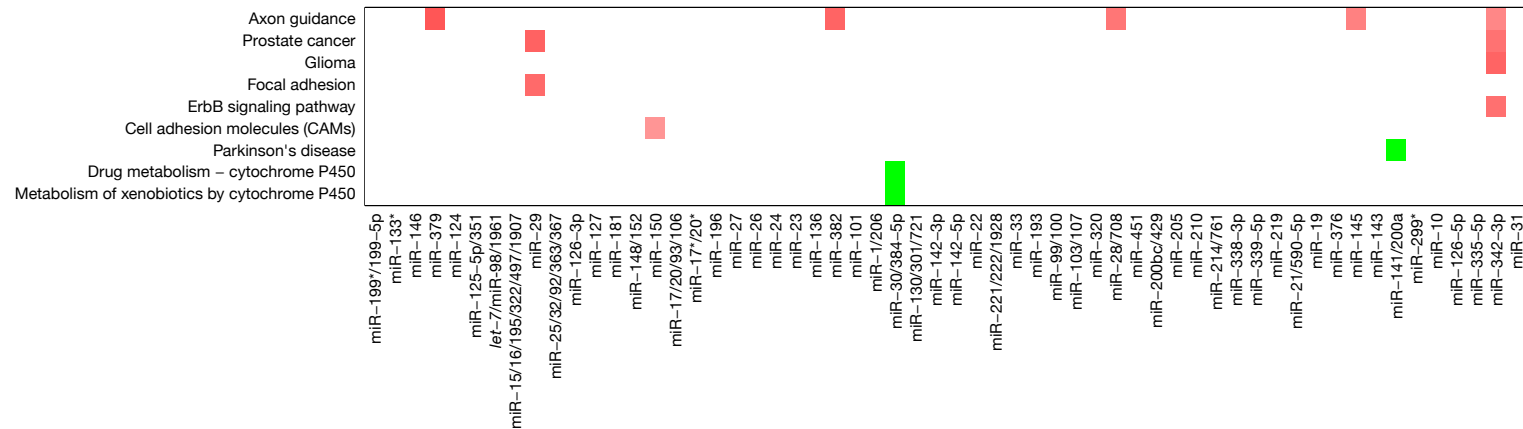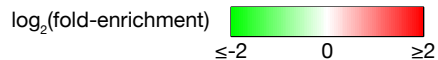

(\*) regulation of nucleobase, nucleoside, nucleotide and nucleic acid metabolic process

Supplement: Additional file 4 — Enrichment and depletion of GO biological processes (a, b) and KEGG pathways (c, d) among predicted miRNA targets. Target genes were defined as genes with at least one 3' UTR match to the seed sequence of one or more miRNAs in a given cluster (a, c) or to individual seed-identical miRNA families (b, d). Log2 fold-enrichments are shown as heatmaps with red and green corresponding to enrichment and depletion, respectively. Significance was assessed by a two-sided Fisher's Exact test. Shown are gene sets with Benjamini-Hochberg corrected P < 0.01 (out of all statistically significant GO terms for a given cluster or miRNA family, only the most specific GO terms were included). [file 1471-2164-10-548-S4.PDF]

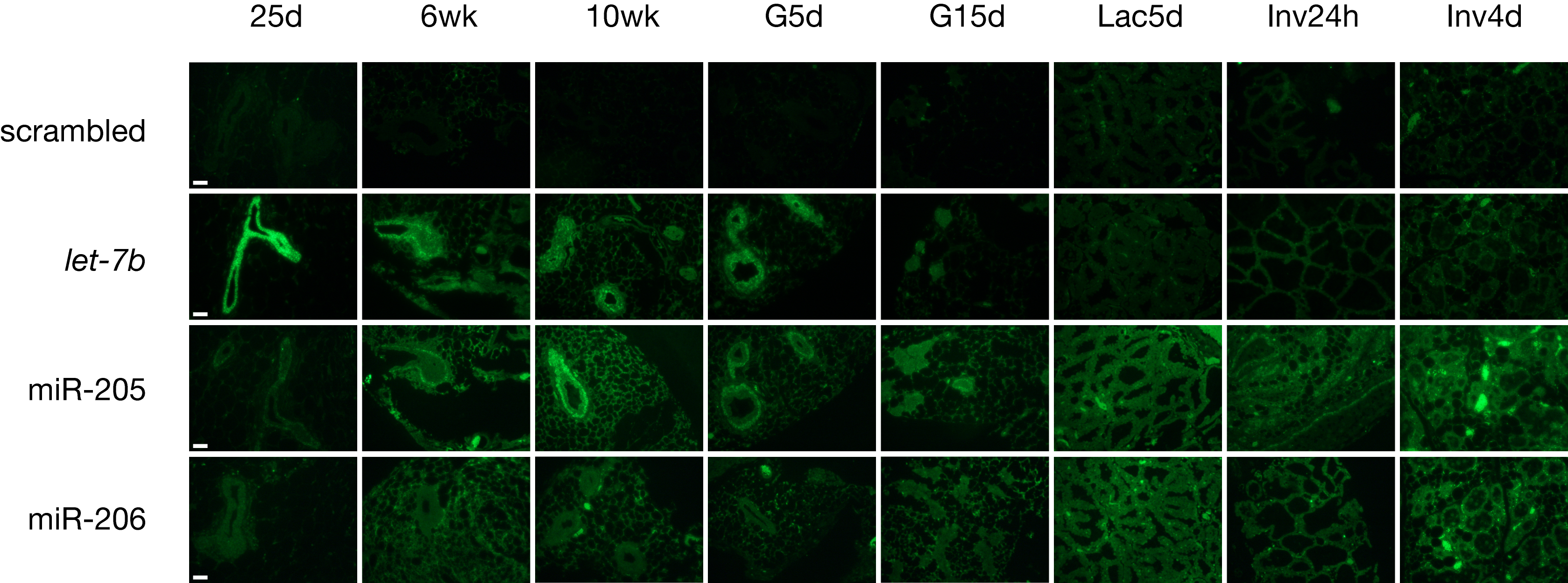

Supplement: Additional file 6 — Higher-magnification images of miRNA in-situ hybridisation. Higher-magnification images of in-situ hybridisation for miRNAs let-7b, miR-205, and miR-206, showing higher expression in the epithelial compared to the stromal cell compartment, and specificity for the luminal and basal epithelial cell layers for let-7b and miR-205, respectively. White scale bars in images for time point 25d indicate 100 microns. [file 1471-2164-10-548-S6.PNG]

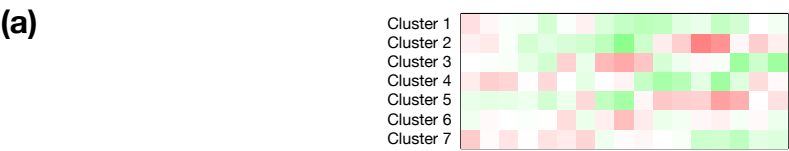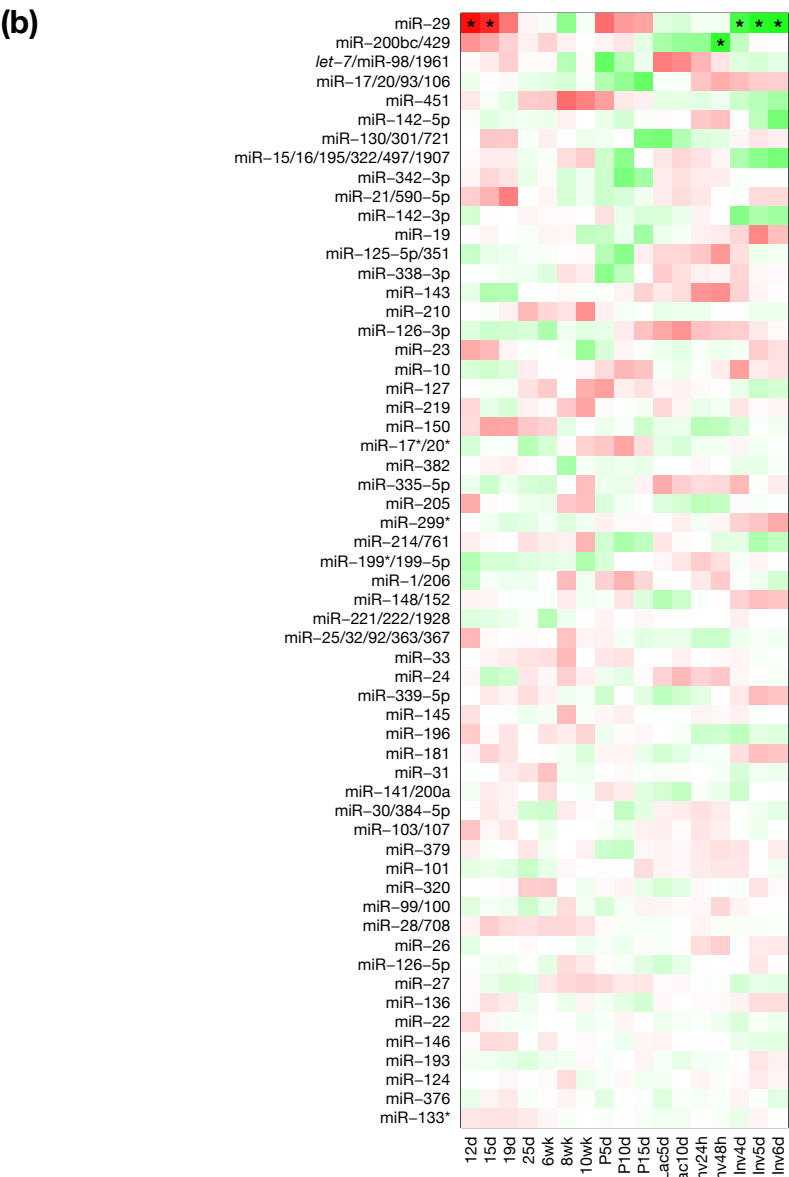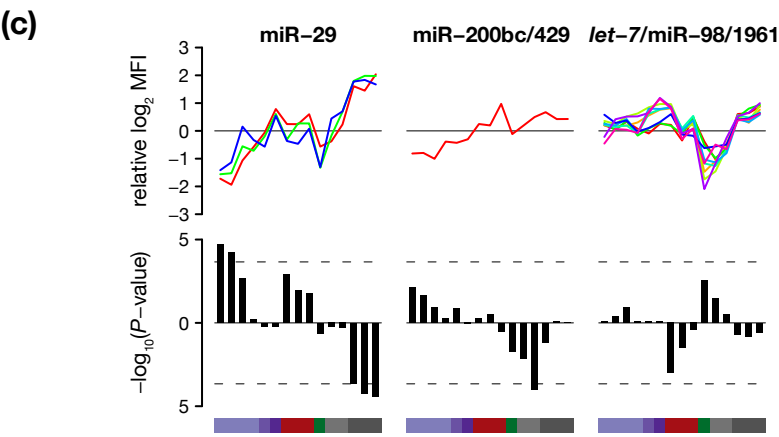

Supplement: Additional file 7 — Changes in the expression of predicted miRNA targets. Negative Log10 transformed two-sided P-values (see Methods) are shown as heatmaps with red and green corresponding to evidence for increased and reduced relative expression levels, respectively. Target genes were defined as genes with at least one 3' UTR match to the seed sequence of one or more miRNAs in a given cluster (a) or to individual seed-identical miRNA families (b). miRNA families were ordered by the evidence for systematic changes in the expression of their targets (based on the minimum observed P-value). Asterisks indicate Benjamini-Hochberg corrected P < 0.05. (c) Greatest evidence for systematic changes in target expression was observed for miRNA families miR-29, miR-200bc/429 and let-7/miR-98/1961. Dashed lines indicate the threshold for Benjamini-Hochberg corrected P-values smaller than 0.05. [file 1471-2164-10-548-S7.PDF]
