# Supplementary material for: Characterisation of microRNA expression in post-natal mouse mammary gland development
Source: BMC Genomics. 2009 Nov 20;10:548. doi: 10.1186/1471-2164-10-548 (PMC2784809; doi:10.1186/1471-2164-10-548)
Supplement: Additional file 2 — Validation of gene (mRNA) expression by qRT-PCR. a. Validation of selected mRNA genes by qRT-PCR using custom designed primers and SYBR Green reporter (left panel), normalising data to the expression values of housekeeping genes beta-actin (Actb) and cyclophilin A (Ppia) (bottom rows). Expression data obtained by Illumina profiling (middle panel) showed good correlation with qRT-PCR data (right panel). b. Detailed description of SYBR Green qPCR according to MIQE guidelines. [file 1471-2164-10-548-S2.PDF]

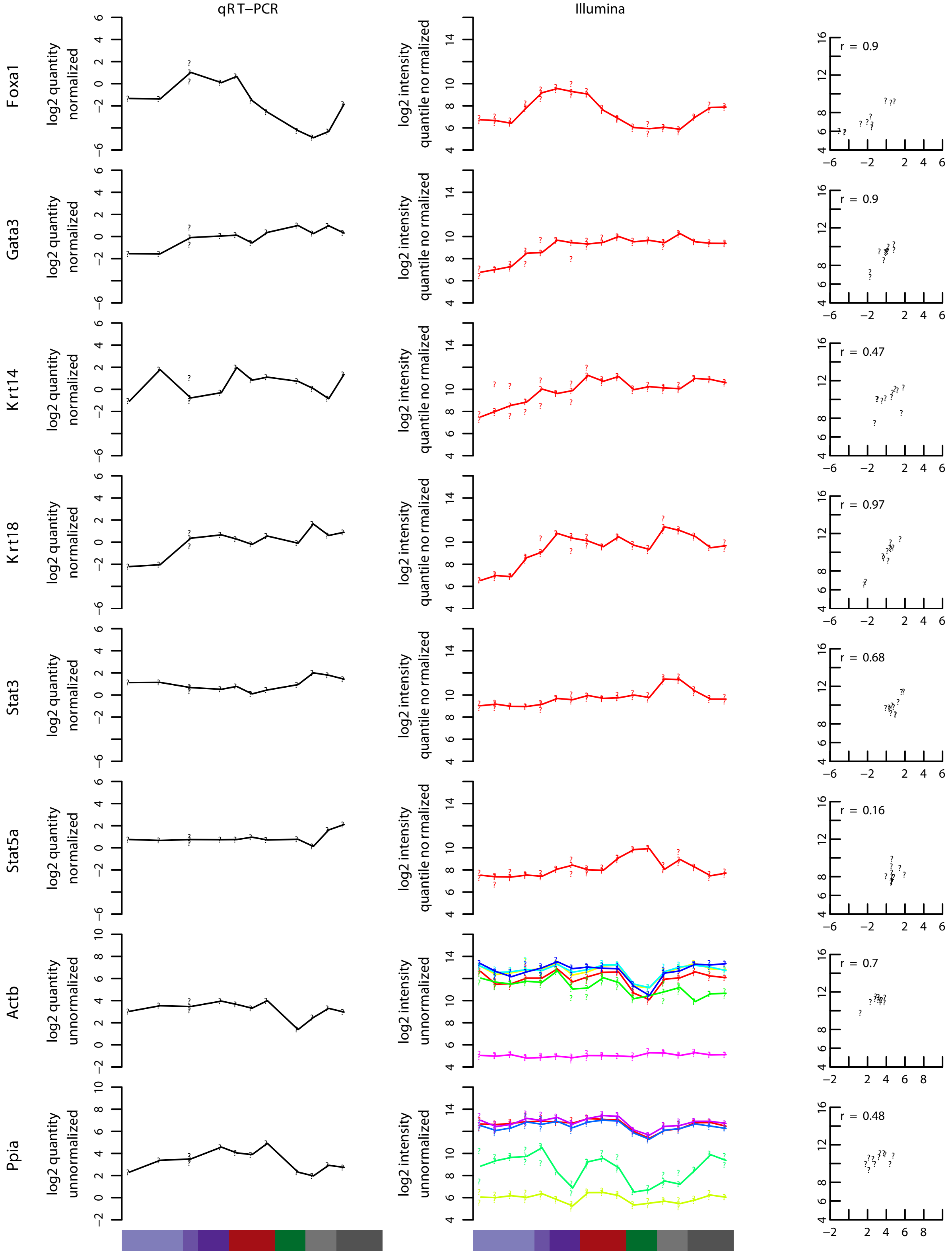
qRT−PCR
Illumina
Foxa1
Gata3
Krt14
Krt18
Stat3
Stat5a
Actb
Ppia
log2 quantity normalized
log2 quantity unnormalized
log2 intensity quantile normalized
log2 intensity unnormalized
r = 0.9
r = 0.9
r = 0.47
r = 0.97
r = 0.68
r = 0.16
r = 0.7
r = 0.48

b. *Experimental design*: Six genes (mRNAs) were selected for validation based on their relevance and previously characterized differential expression during mouse mammary gland development. These included Foxa1, Gata3, Krt14, Krt18, Stat3, and Stat5a. In addition, beta-actin (Actb) and cyclophilin A (Ppia) were used as reference genes for normalisation. Twenty-two out of 57 samples assessed by bead-based microarrays, including one biological replicate for all 18 time points, and all three replicates for time points 6wk and 12wk, were selected for qRT-PCR validation. In addition, all samples were combined into a sample pool using equal mRNA quantities per sample, to be used as input controls and for serial dilutions.

Sample processing and RNA extraction are described in detail in the methods section of the main text.

Reverse transcription (RT) reaction: The RT reaction was prepared using a standard input of 1µg of total RNA in a reaction volume of 20µl, including 2.5µM random hexamers, 1xRT buffer (Applied Biosystems), 5mM MgCl2, 4mM dNTPs, 0.5U/µl RNAse inhibitors, and 2U/µl reverse transcriptase (all reagents purchased from Applied Biosystems). As controls, the RT reaction was once prepared without sample input and once without reverse transcriptase, to control for potential contamination. The reaction was carried out for 10min at 25°C, for 30min at 48°C, for 5 min at 95°C, with a final hold step at 4°C (min. 10min at 4°C).

qPCR protocol:

All RT-PCR primers were designed to span exons using the Primer3 software (Whitehead Institute for Biomedical Researc; http://primer3.sourceforge.net/), and subsequent dissociation curve analysis was performed to confirm that each pair of primers produced a single product. Primer sequences were as follows: Gata-3 (F: CTTATCAAGCCCAAGCGAAG; R: CATTAGCGTTCCTCCTCCAG), Stat3 (F: CAGGGTGTCAGATCACATGG; R: TAGCCAGACCCAGAAGGAGA), Stat5a (F: CTGAAGGAAGCAGAGGGATG; R: TGCAAAAACCAAACCAACAA), Foxa1 (F: CATGAGAGCAACGACTGGAA; R: TGTTGCTGACAGGGACAGAG), Krt1-14 (F: GCTCTTGTGGTATCGGTGGT; R: GAGGAGAAGCGAGAGGAGGT), and Krt1-18 (CGAGGCACTCAAGGAAGAAC; R: AATCTGGGCTTCCAGACCTT), Actb (F: GATCATTGCTCCTCCTGAGC; R: ACATCTGCTGGAAGGTGGAC), Ppia (F: GCATACAGGTCCTGGCATCT; R: ATCCAGCCATTCAGTCTTGG).

The qPCR reaction was prepared using a total reaction volume of 20 µl including 7µl of SYBR green PCR Master Mix, containing SYBR Green 1 Dye, AmpliTaq Gold DNA Polymerase (Applied Biosystems concentrations of Mg++, dNTP, and polymerase not disclosed), 1µl each of forward and reverse primer (100nM), and a standard input of 10ng

cDNA. The qPCR reaction was carried out on the ABI Prism 7900HT sequence detection system (Applied Biosystems) for 12min at 95°C, followed by 40 cycles with 20sec at 95°C, 20sec at 54°C and 30sec at 72°C per cycle. A final dissociation step using a ramp from 60-95°C was included to confirm a single PCR product.

Serial dilutions were prepared by using the sample pool consisting of equal amounts of all individual samples analysed. Five serial dilutions, using 2x, 1x, 0.25x, 0.0625x, and 0.0156x of the standard input into the PCR reaction were analysed in triplicates for subsequent calculation of a standard curve.

Data analysis: For each miRNA assay, all samples, no-template-controls (NTC) were analysed in triplicates in a single run on one plate, including serial dilutions for a standard curve for every assay. The threshold value for fluorescence detection was manually selected to be within the exponential amplification phase of all samples and serial dilutions. The SDS software (version 2.3, Applied Biosystems) was used to automatically generate a standard curve. After visual inspection of the standard curve, the manual removal of up to three out of 18 single data points from different dilutions was allowed to remove outliers. Subsequently, the SDS software was used to automatically calculate the slope (range -3.4 to -3.8), y-intercept (range 22.0 to 30.3), and r^2 (≥0.99) of the standard curve, and subsequently the threshold cycle (C_T) and relative quantity of each sample. Using SDS software, the data was then exported into a Microsoft Excel file for further analysis.

The relative mRNA expression throughout the 18-point developmental time course as determined by qPCR was then compared to the relative expression determined by microarray using custom scripts in the R programming environment.
