# Supplementary material for: Characterisation of microRNA expression in post-natal mouse mammary gland development
Source: BMC Genomics. 2009 Nov 20;10:548. doi: 10.1186/1471-2164-10-548 (PMC2784809; doi:10.1186/1471-2164-10-548)
Supplement: Additional file 5 — miRNA expression and genomic location. Heatmap as in main text Figure 3b with miRNAs in individual clusters reordered according to their genomic loci. Brackets in the right-hand margin indicate genomic clusters. A mature miRNA was assigned to a genomic cluster if its locus was situated within 50 kb of another cluster member (genomic clusters in distinct regions of the genome that shared a common mature miRNA were merged). [file 1471-2164-10-548-S5.PDF]

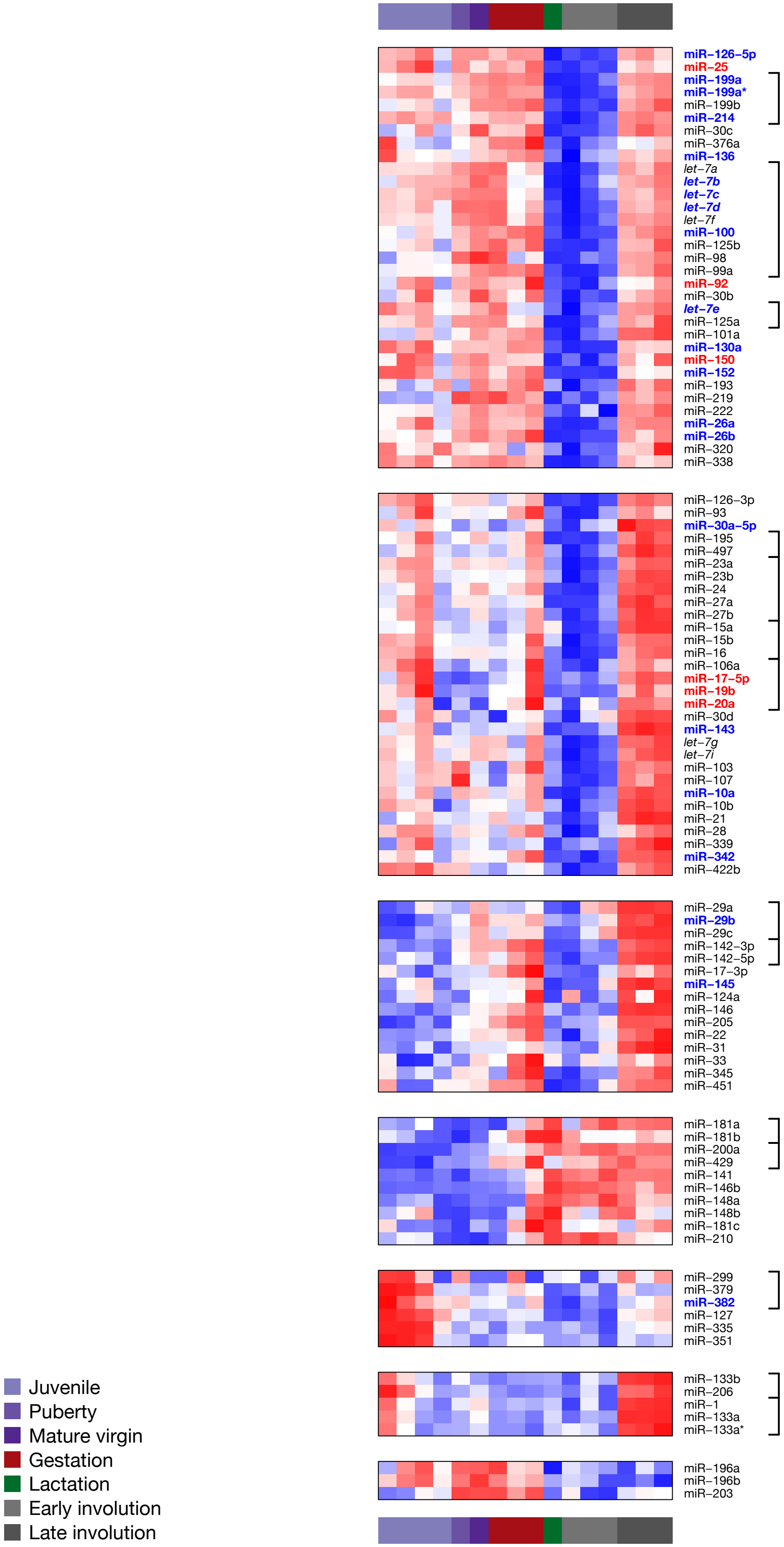
miR−126−5p
miR−25
miR−199a
miR−199a*
miR−199b
miR−214
miR−30c
miR−376a
miR−136
let−7a
let−7b
let−7c
let−7d
let−7f
miR−100
miR−125b
miR−98
miR−99a
miR−92
miR−30b
let−7e
miR−125a
miR−101a
miR−130a
miR−150
miR−152
miR−193
miR−219
miR−222
miR−26a
miR−26b
miR−320
miR−338
miR−126−3p
miR−93
miR−30a−5p
miR−195
miR−497
miR−23a
miR−23b
miR−24
miR−27a
miR−27b
miR−15a
miR−15b
miR−16
miR−106a
miR−17−5p
miR−19b
miR−20a
miR−30d
miR−143
let−7g
let−7i
miR−103
miR−107
miR−10a
miR−10b
miR−21
miR−28
miR−339
miR−342
miR−422b
miR−29a
miR−29b
miR−29c
miR−142−3p
miR−142−5p
miR−17−3p
miR−145
miR−124a
miR−146
miR−205
miR−22
miR−31
miR−33
miR−345
miR−451
miR−181a
miR−181b
miR−200a
miR−429
miR−141
miR−146b
miR−148a
miR−148b
miR−181c
miR−210
miR−299
miR−379
miR−382
miR−127
miR−335
miR−351
miR−133b
miR−206
miR−1
miR−133a
miR−133a*
miR−196a
miR−196b
miR−203
Juvenile
Puberty
Mature virgin
Gestation
Lactation
Early involution
Late involution
