# Supplementary material for: Characterisation of microRNA expression in post-natal mouse mammary gland development
Source: BMC Genomics. 2009 Nov 20;10:548. doi: 10.1186/1471-2164-10-548 (PMC2784809; doi:10.1186/1471-2164-10-548)
Supplement: Additional file 8 — Validation of miRNA expression by qRT-PCR. a. Validation of selected miRNA genes by qRT-PCR using TaqMan miRNA Assays (left panel). qRT-PCR measurements were normalised to U6 small nuclear RNA (U6sRNA; bottom row). Expression data obtained by Luminex bead-based profiling (middle panel) showed good correlation with qRT-PCR data (right panel). b. Detailed description of TaqMan qPCR according to MIQE guidelines. [file 1471-2164-10-548-S8.PDF]

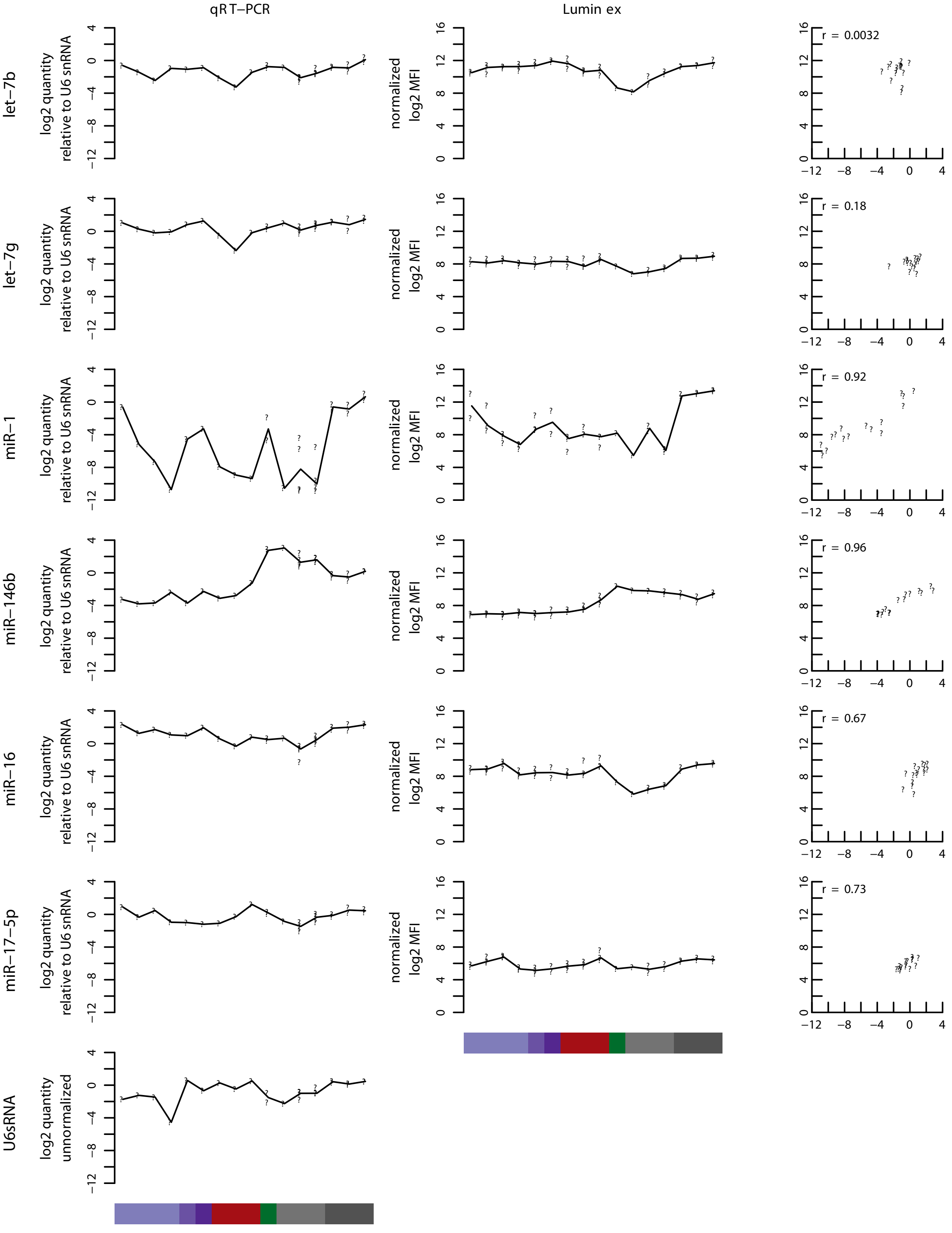

qRT−PCR
Lumin ex
let−7b
let−7g
miR−1
miR−146b
miR−16
miR−17−5p
U6sRNA
log2 quantity relative to U6 snRNA
log2 quantity unnormalized
normalized log2 MFI
r = 0.0032
r = 0.18
r = 0.92
r = 0.96
r = 0.67
r = 0.73

b. *Experimental design*: Six miRNAs were randomly selected for validation by qRT-PCR, including let-7b, let-7g, miR-1, miR-146, miR-16, and miR-17-5p. In addition, U6 small nuclear RNA was used as reference for normalization. Thirty out of 57 samples assessed by bead-based microarrays, including all 18 time points and 1 to 4 biological replicates per time point, were selected for qRT-PCR validation. In addition, for each miRNA to be analysed, all samples were combined into 2 or 3 sample pools with high, (intermediate), and low expression based on microarray data to be used as input controls and for serial dilutions.

Sample processing and RNA extraction are described in detail in the methods section of the main text.

Reverse transcription (RT) reaction: The RT reaction was performed according to manufacturer’s instructions, with the modification of using a total reaction volume of 10µl instead of 15µl. The standard input into the RT reaction was 4ng of total RNA (manufacturer’s recommendation 1-10ng) in a total reaction volume of 10µl. Two to three sample pools, with low and high expression of the respective miRNA based on microarray data, were used as input controls into the RT reaction, using standard input (4ng), 0.5x standard (2ng) and 1.5x standard input (6ng) to confirm that the RT reaction was carried out within its linear range without inhibition. The RT reaction was prepared separately for each miRNA assay, using a miRNA-specific looped primer (provided by manufacturer, sequence not disclosed) and MultiScribe Reverse Transcriptase (provided by manufacturer) at a concentration of 3.3U/µl in the final reaction volume. As controls, for each miRNA assay the RT reaction was once prepared without sample input and once without reverse transcriptase, to control for potential contamination. The RT reaction was carried out for 30min at 16°C, 30min at 42°C, 5min at 85°C, with a final hold step at 4°C C (min. 10min at 4°C).

qPCR protocol: The qPCR reaction was performed according to the manufacturer’s protocol, with the modification of using a total reaction volume of 10µl instead of 20µl. The qPCR reaction was prepared using ‘TaqMan 2x PCR MasterMix No AmpErase UNG’ containing AmpliTaq Gold DNA Polymerase (purchased separately from manufacturer, concentrations of Mg++, dNTP, and polymerase not disclosed), a miRNA-specific forward primer and a universal reverse primer complementary to the loop sequence of the RT primers (provided by manufacturer, sequences not disclosed), and the respective RT products in a 1 in 15 dilution. The qPCR reaction was carried out on the ABI Prism 7900HT sequence detection system (Applied Biosystems), using 9600 emulation run mode, for 10min at 95°C, followed by 40 cycles with 15sec at 95°C and 60sec at 60°C per cycle.

Serial dilutions were prepared by using the sample pool with high expression of the respective miRNA based on microarray data, to ensure that quantification could be performed within the linear dynamic range of the amplification reaction for all samples. Six serial dilutions, using 3x, 1x, 0.33x, 0.11x, 0.037x, and 0.012x of the standard input into the PCR reaction were analysed in triplicates for subsequent calculation of a standard curve.

Data analysis: For each miRNA assay, all samples, no-template-controls (NTC) were analysed in triplicates in a single run on one plate, including serial dilutions for a standard curve for every assay. The threshold value for fluorescence detection was manually selected to be within the exponential amplification phase of all samples and serial dilutions. The SDS software (version 2.3, Applied Biosystems) was used to automatically generate a standard curve. After visual inspection of the standard curve, the manual removal of up to three out of 18 single data points from different dilutions was allowed to remove outliers. Subsequently, the SDS software was used to automatically calculate the slope (range -2.0 to -3.3), y-intercept (range 21.2 to 30.0), and r^2 (range 0.90 to 0.98) of the standard curve, and subsequently the threshold cycle (C_T) and relative quantity of each sample. Using SDS software, the data was then exported into a Microsoft Excel file for further analysis.

For each assay and all samples, the standard deviation and the percentage standard deviation (SD) of the quantity was calculated among each triplicate. Overall, the %SD ranged between 8% and 15%. Those triplicates with a percentage standard deviation of ≥ 20% were identified, and manual removal of one out of three replicates was allowed if a clear outlier could be identified by visual inspection. Overall, a mean of 8.1 outliers out of 135 replicates per assay were removed manually. After removal of outliers, the mean value of the remaining two or three replicates was used for further analysis.

The relative miRNA expression throughout the 18-point developmental time course as determined by qPCR was then compared to the relative expression determined by microarray using custom scripts in the R programming environment.
